# Arabic emphatic consonants as produced by English speakers: An acoustic study

**DOI:** 10.1016/j.heliyon.2023.e13401

**Published:** 2023-02-02

**Authors:** Hesham Aldamen, Mutasim Al-Deaibes

**Affiliations:** a Yarmouk University, Irbid, Jordan; b Khalifa University Abu Dhabi, United Arab Emirates

**Keywords:** Emphasis, L2 Arabic, Second language acquisition, VOT, L2 phonetics

## Abstract

This study examines the production of emphatic consonants as produced by American L2 learners of Arabic. To this end, 19 participants, 5 native speakers and 14 L2 learners, participated in a production experiment in which they produced monosyllabic CVC pairs that were contrasted in terms of whether the initial consonant was plain or emphatic. The acoustic parameters that were investigated are VOT of voiceless stops, COG of fricatives, and the first three formant frequencies of the target vowels. The results of the native speakers showed that VOT is a reliable acoustic correlate of emphasis in MSA. The results also showed that vowels in the emphatic context have higher F1 and F3 and lower F2. The results showed that the L2 learners produced comparable VOT values to those of native Arabic speakers. Further, L2 learners produced a significantly lower F2 of the vowels in the emphatic context than that in the plain context. Proficiency in Arabic played a role on the F2 measure; the intermediate learners tended to be more native-like than the beginning learners. As for F3, the results of the L2 learners unexpectedly showed that the beginning learners produced a higher F3 in the context of fricatives only. This suggests that the relationship between emphasis and proficiency depends on whether the preceding consonant is a stop or fricative.

## 1 Introduction and background

Emphasis is a phonetic and phonemic feature of Semitic languages such as Arabic, Modern Hebrew, and Ethiopic-Ge’ez. For example, the words /tab/ “he repented” and **/**tˤab/ “he recovered” contrast in terms of whether the first consonant is plain or emphatic. According to Ref. [1], the primary constriction usually occurs in the dental or alveolar region, and the secondary constriction occurs in the back of the vocal tract. Although much phonetic research has been done on emphasis in Arabic (e.g., Refs. [[2], [3], [4], [5], [6], [7], [8], [9], [10]]), there is still no consensus on the precise nature of the secondary constriction occurring in the back of the vocal tract. This might be due to the different dialects that have been studied, methodological or laboratorial limitations, and the articulatory complexity of emphasis. To examine the precise nature of the secondary constriction occurring in the back of the vocal tract during the production of emphatics [11], conducted both a physiological and acoustic study of the pharyngeal and emphatic consonants in four Arabic dialects (those spoken in Beirut, Nablus, Baghdad and Beer-Zeit). The findings showed that pharyngeal and emphatic consonants exhibit the same articulatory gestures in the pharynx. The epiglottis makes a constriction against the pharyngeal wall, and the tongue root retracts backwards. However, in the production of emphatic consonants, the constriction is less than that involved in the production of pharyngeal consonants. This may be due to the primary (not secondary) pharyngeal constriction in the production of pharyngeals. In their analysis of the acoustic correlates of emphasis, the authors compared the formant values of vowels in the context of plain and emphatic consonants. According to their acoustic results, the pharyngeal constriction in the production of emphatic consonants was accompanied by a lowered second formant frequency (F2) and a slightly raised first formant frequency (F1) of the adjacent vowel.

Another definition of emphasis that deviated from the one discussed above was provided by Ref. [12] who argued that emphatic sounds are uvularized, not pharyngealized (or velarized). McCarthy’s argument is supported by Zawaydeh’s [13] study of the phonology and phonetics of gutturals of Jordanian Arabic spoken in Amman. On the other hand [15], conducted a videofluoroscopic study of the emphatic consonants in Jordanian Arabic and argued against Zawaydeh’s [13,14] results. The results showed that emphatic consonants are produced with pharyngealization resulting in the tongue root moving back into the oropharynx, causing the elevation of the hyoid bone, and raising of the larynx. It is worth mentioning here that the Arabic emphatic consonants consist of two fricatives and two stops. The emphatic fricatives are the voiced interdental /ðˤ/ and the voiceless denti-alveolar /sˤ/, and the emphatic stops are the voiced denti-alveolar /dˤ/ and the voiceless denti-alveolar /tˤ/. The Arabic plain consonants, i.e., / ð/, /s/, /d/, and /t/ are articulatory similar to their English counterparts and hence do not pose any articulatory problems for English-speaking learners of Arabic. The full consonantal and vocalic inventories of Standard Arabic are laid out in Table 1, Table 2.

**Table 1:** Consonantal inventory of Modern Standard Arabic (MSA) (Adapted from Ref. [6]).

	Labial	Labio-dental	Inter-dental	Alveolar	Palatao- Alveolar	Palatal	Velar	Pharyngeal	Uvular	Glottal
Plosive	b			t d tˤ dˤ			K		q	ʔ
Nasal	m			n						
Trill				r						
Fricative		f	θ ð ðˤ	s z sˤ	ʃ		x ɣ	ħ ʕ		h
Affricate					ʤ					
Lateral				l						
Glide	w					j

**Table 2:** Vocalic inventory of MSA (Adapted from Ref. [6]).

Front	Back
High i/i:	u/u:
Low a/a:	

### 1.1 Acoustic studies of emphasis

Much research in comparable milieus were conducted on the acoustic correlates of emphasis in Arabic (see Refs. [[2], [3], [4], [5], [6], [7], [8], [9], [10]], inter alia). Each study concentrated on a specific dialect of the Arabic language. It is worth mentioning here that the consensus of all the acoustic studies that investigated the acoustic correlates of emphasis in Arabic is that F2 of vowels in the emphatic environments significantly lowers.

Card [2] conducted a phonetic and phonological investigation of emphasis in Palestinian Arabic. The results of the acoustic experiment showed that lowering F2 was the only major acoustic correlate for emphasis in Palestinian Arabic regardless of whether the emphatic consonant was in word-initial or word-final position. F1 and the third formant frequency (F3) were not affected by emphasis. According to Ref. [2], emphasis mostly affected the low and back vowels, i.e., /a/ and /u/, and their long versions /a:/ and /u: / as well as the long back vowel /o:/. However [2], did not mention where in the vowel she measured the formant frequencies.

In an acoustic investigation of another dialect of Arabic [3], examined the acoustic correlates of emphasis in Egyptian Arabic spoken in Alexandria [3]. used monosyllabic and disyllabic word pairs. F1 and F2 were measured at the onset and midpoint of the vowels. The results showed that there was no significant difference in F1 values in the emphatic and non-emphatic environments. As for F2, it was significantly lowered in the emphatic environment at the onset and midpoint of the vowels. The vowels that were mostly affected by emphasis in terms of lowering of F2 were the low central vowels /a/ and /a:/. These results are compatible with those of [2] in that F2 lowers significantly in the environment of emphatic sounds, but not F1. However [2], reported that the vowels /u/ and /a/ mostly affected by emphasis.

In a more recent investigation [4,25], examined the acoustic correlates of emphasis in the northern dialect of Jordanian Arabic. The results showed again that F2 of the vowel was significantly lowered in the emphatic context. More converging evidence that supported the findings of the previous studies was provided by Ref. [16] who addressed the acoustic correlates of /tˤ/ in the speech of males and females [16]. reported that F2 of vowels was significantly lowered in the emphatic environment of /tˤ/, and F1 was significantly raised. F1 and F2 were measured at the onset of the vowel. As for voice onset time (VOT), /t/ has a significantly longer VOT than /tˤ/.

Al-Masri [4] addressed the acoustic and perceptual correlates of emphasis in urban Jordanian Arabic. The findings showed that F1 was raised in emphatic contexts; however, the effect of emphasis was gradient. Concerning F2, the results showed that it was lowered in emphatic environments at all three vowel positions. The results also showed that the high back vowels /u/ and /u:/ are less affected by emphasis than high front and low front vowels /i/ and /i:/ and /a/ and /a:/, respectively; According to Ref. [4], the low front vowels /a/and /a:/ showed the largest drop of F2 at the midpoint.^1^ This supported [3]’s findings in that high back vowels are among the least affected by emphasis. This also corroborated the results of [2] in that the low vowels are among the most affected by emphasis. In terms of F3, the results showed that it was raised in emphatic environments, the center of gravity (COG) for the emphatic stops was higher than that for the non-emphatic stops, and the emphatic fricatives had a lower COG than the plain ones.

In another study [17], examined the effects of gender on the production of emphasis in Jordanian Arabic. The results showed that VOT of the emphatic plosives was shorter than that of the plain plosives. As for F1, the results showed that it was raised in the emphatic environment at the beginning and midpoint of the vowel. Concerning F2, it was lowered in the emphatic environment in all three vowel positions. F3 was raised in the emphatic context at the beginning and end of the vowel. F2 lowering provided support for the claim that emphasis spread is gradient (e.g., Refs. [18,27]). As in most of the studies, the low vowels /a/ and /a:/ were the most affected by F2 lowering at the midpoint.

Similarly, in their recent phonetic study of two Jordanian Arabic dialects [7], dealt with the acoustics of emphasis in urban and rural Jordanian Arabic. The results showed that F1 and F3 were raised, and F2 was lowered in the emphatic environment.

In sum, most previous acoustic studies on emphasis showed that the first three formant frequencies (F1–F3), especially F2, and VOT are reliable acoustic parameters for emphasis.

### 1.2 Second language (L2) speech production studies

[19] conducted a phonetic study to investigate the production of Palestinian-accented MSA vowels by English L2 learners and heritage speakers of Arabic. For the purpose of this study, the focus will be on only the production of plain and emphatic Arabic vowels. The results showed that F1 of the vowels /i/, /i:/, /u/, and /u:/ was slightly raised in the emphatic context across the three groups; the difference, however, did not reach significance. F1 for the low vowels /a/ and /a:/ was slightly lower in the emphatic environment. As for F2, it was significantly lower in the emphatic environment across the three groups. However, there was no interaction between language experience and vowel pharyngealization. In other words, the three groups patterned the same.

Considering the findings of the previous study, the more experienced L2 learners are expected to be more native-like in their production of emphasis than the less experienced L2 learners. In other words, the more experienced L2 learners’ acoustic correlates to emphasis are expected to be closer to those of native speakers.

### 1.3 The Speech Learning Model

The Speech Learning Model (SLM) was developed by Ref. [20] as a model of L2 speech acquisition [20]. reported that SLM attempts to model the level of success highly-experienced L2 learners will achieve in the perception and production of L2 sounds. Therefore, SLM makes predictions about how L2 learners will perform in L2 speech perception and production based on the perceived phonetic distance that exists between the L1 and L2 sounds. The SLM accounts for L2 speech learning, and how it affects the phonetic categories formed during L1 acquisition. The predictions made by SLM regarding the degree of accuracy with which highly experienced learners will perceive and produce L2 sounds can be tested empirically. The SLM posits that the speech learning mechanisms (e.g., the ability to form phonetic categories) that are employed in the acquisition of L1 can also be exploited in L2 acquisition. It also makes the hypothesis that L2 learners can form new phonetic categories depending on whether they detect adequate phonetic dissimilarities existing between L1 and L2 sounds; discernibility of phonetic differences between L2 and L1 sounds depends on the perceived phonetic distance existing among them. According to the SLM, establishing new phonetic categories will enable L2 learners to perceive and produce L2 sounds in a native-like fashion with significantly less interference from the L1.

### 1.4 Focus of the present study

This study aims at investigating the production of emphasis by native American-English speakers who are L2 learners of Arabic. To the best of our knowledge, only [19] examined the production of emphasis by American L2 learners of Arabic. The acoustic cues that were examined in that study were F1 and F2 of the vowels in the context of emphatic and plain consonants. Moreover, only language experience and vowel pharyngealization were the independent variables. However, the present study is more comprehensive in terms of the acoustic correlates to be examined; in addition to measuring F1–F3 values in emphatic and plain environments, the characteristics of the consonants are also examined by measuring the VOT of the voiceless emphatic and plain stops and the spectral mean of emphatic and plain fricatives. The findings of the current study will be interpreted in light of the predictions of SLM. The reason why SLM is used over other models is because it is a model that accounts for both L2 production and perception and explicitly draws a link between them. It also focuses on learners who acquired the L2 in a naturalistic context and have reached their ultimate attainment.

The research questions of the current study are:

1. Do native speakers and L2 learners maintain a contrast between plain and emphatic consonants in the following acoustic parameters: VOT of voiceless stops, COG of fricatives, and F1, F2, and F3 of the following vowels?
2. Is the production of emphasis influenced by experience with Arabic?
3. Is learners’ production of the vowels in the emphatic context based on already existing English phonetic categories or on new phonetic ones?

## 2 Methodology

### 2.1 Participants

The participants in this experiment were 14 learners of Arabic who had been studying Arabic for 1–4^2^ years and 5 native speakers of Arabic. The participants were 18–33 years old. The native Arabic speakers were recruited from the local community in the city of Lawrence, Kansas. They were all native speakers of Saudi Arabic who reported that Arabic was their dominant language at the time of the study. All the American participants were students at the University of Kansas who reported normal speech and hearing. The participants were asked to fill out a background language questionnaire (see Appendix C). All participants voluntarily participated in the study.

### 2.2 Stimulus materials

The production stimuli of this study consisted of a word list of 24 monosyllabic minimal pairs. All of them contained one of the long and short vowels /i:/, /a:/, /u:/, /i/, /a/, /u /. The minimal pairs were contrasted in terms of whether the initial consonant was emphatic or plain. The target consonants in this study were /ð/, /ðˤ/, /t/, /tˤ/, /d/, /dˤ/, and /s/, /sˤ/ (e.g., /ta:b/ and / tˤa:b/. The minimal pairs that were used in this experiment are taken from MSA. Nonwords were also used; all of the nonwords obeyed the phonological phonotactics of MSA. The list of tokens is provided in Appendix B.

### 2.3 Procedures

Prior to data collection, a consent form was handed to the participants to seek their permission to record the stimuli and to explain the purpose of the study. Upon receiving the approval from Research Ethics Committee at Kansas University, the participants were provided with a written list of the minimal pairs used in this study. They were asked to read the randomized minimal pairs at a normal rate. Each stimulus was read once. The words were presented to participants in the Arabic language orthography supplemented with diacritic markings. The target word pairs were recorded in the carrier phrase [ˈʔiħki___ˈmar:ah] (“Say____once”). The recordings were performed in the anechoic chamber at the University of Kansas with a Marantz PMD-671 solid state recorder and an Electro Voice 767 microphone.

### 2.4 Acoustic measurements

Praat (Version 4.6.09) [21] was used to perform the acoustic measurements in this study. These measurements consisted of VOT of the voiceless plain and emphatic stops, the spectral mean of plain and emphatic fricatives, and the first 3 formant frequencies (F1–F3) of the vowels following the target plain and emphatic consonants. The VOT of voiceless plain and emphatic stops was measured as the duration between the release of the consonant (burst) and the onset of voicing of the following vowel. As in Ref. [1], the spectral mean was measured over a 20-ms Hamming window in the middle of the friction. Formant frequency measures (F1–F3) were taken from LPC spectra calculated over a 20-ms Hamming window at vowel midpoint.

## 3 Results

### 3.1 Voice onset time for voiceless stops

**Fig. 1:**
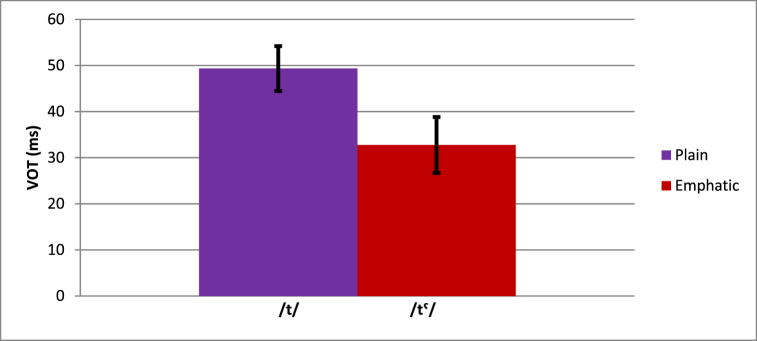
4: Difference in VOT for plain and emphatic /t/.

The VOT results were analyzed using a mixed analysis of variance (ANOVA) with the within-subjects factors Emphasis (2 levels: plain, emphatic), Vowel Length (2 levels: long, short), Vowel Context (3 levels: /a/, /i/, /u/), and the between-subjects factor Proficiency (3 levels: native, intermediate, beginner). For this and the following analyses, the Greenhouse-Geisser correction was used for tests of effects with more than one degree of freedom in the numerator. The ANOVA revealed significant main effects of Emphasis (*F* (1, 16) = 28.97, *p* < .001) and Vowel Length (*F* (1, 16) = 23.57, *p* < .001), as well as a marginal Vowel Length * Proficiency interaction (*F* (2, 16) = 3.34, *p* = .061). The effect of Emphasis indicated that VOT was longer for plain (49 ms) than emphatic (33 ms) consonants (see Fig. 1). The effect of Vowel Length indicated that VOT was longer for consonants preceding long vowels (48 ms) than those preceding short vowels (34 ms). The Vowel Length * Proficiency interaction indicated that the difference in VOT for stops preceding long vowels compared to short vowels was small for native speakers (3 ms), but larger for intermediate (17 ms) and beginning learners (19 ms). Post-hoc comparisons of the vowel length effects (the difference scores for the *long* condition minus the *short* condition) revealed that the effect for native speakers was smaller than for intermediate learners (*t* (6.6) = −2.09, *p* = .077) and beginners (*t* (8.23) = −4.91, *p* = .001), but that the effect did not significantly differ between intermediate learners and beginners (*t* (8.75) = 0.47, *p* = .652).^3^

Although the Emphasis * Proficiency interaction did not approach significance, the effect of Emphasis for intermediate speakers was numerically more native-like than for beginners: native speakers showed an 18.2 ms difference between plain and emphatic consonants, intermediate learners showed a 22.88 ms difference, and beginners showed a 9.12 ms difference.

### 3.2 Center of gravity for fricatives

COGs were analyzed using a mixed analysis of variance (ANOVA) with the within-subjects factors Emphasis (2 levels: plain, emphatic), Vowel Length (2 levels: long, short), Vowel Context (3 levels: /i/, /u/, /a/), and Voicing (2 levels: voiced, voiceless), and the between-subjects factor Proficiency (3 levels: native, intermediate, beginner). The ANOVA revealed a significant effect of Vowel Context (*F* (2, 32) = 3.62, *p* = .047), indicating that COG was significantly affected by whether the fricative preceded /i/ (3760 Hz), /u/ (3554 Hz), or /a/ (3680 Hz). There was also a significant effect of Voicing (*F* (1, 16) = 1854.58, *p* < .001), indicating that COG was substantially higher in voiceless (6983 Hz) than voiced fricatives (347 Hz). Finally, there was a significant Vowel * Voicing interaction (*F* (2,32) = 5.64, *p* = .014), indicating that the difference in COG between voiceless and voiced fricatives was somewhat smaller before /u/ (6322 Hz) than before /a/ (6768 Hz) and /i/ (6819 Hz). No interactions between Emphasis, Proficiency, and other variables reached significance (*F*s < 1.88, *p*s > .185), indicating that L2 learners did not differ from native speakers in terms of their production of COG.

Although the Emphasis * Proficiency interaction did not approach significance, the non-significant effect of Emphasis for intermediate speakers was numerically more native-like than for beginners: native speakers showed a 147.25 Hz difference between plain and emphatic fricatives, intermediate learners showed a −78.8 Hz difference, and beginners showed a −93.9 Hz difference.

### 3.3 First formant frequency (F1)

F1 was analyzed using a mixed analysis of variance (ANOVA) with the within-subjects factors Emphasis (2 levels: plain, emphatic), Vowel Length (2 levels: long, short), Vowel Context (3 levels: /a/, /i/, /u/), Manner (2: stop, fricative), and Voicing (2 levels: voiced, voiceless), and the between-subjects factor Proficiency (3 levels: native, intermediate, beginner). The results of the omnibus ANOVA are shown in Table 3 in Appendix A.

**Fig. 2:**
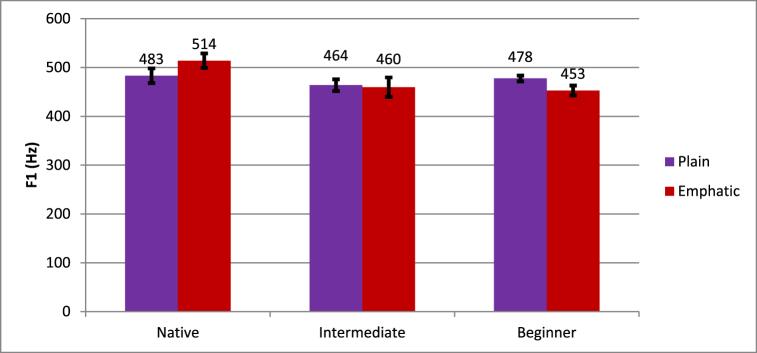
Differences in plain and emphatic F1 across levels of proficiency.

The Emphasis * Proficiency interaction reached significance. The nature of this interaction is shown in Fig. 2, indicating that while native speakers showed higher F1 for emphatic (514 Hz) than plain (483 Hz) tokens, learners showed the opposite effect. Beginners showed lower F1 for emphatic (453 Hz) than plain (478 Hz), and the intermediate learners also showed lower F1 for emphatic (460 Hz) than plain (464 Hz).

To test how different each group of speakers was from native speakers, difference scores were calculated according to the procedure described above. An independent samples *t*-test showed that the difference between beginners and intermediate learners from native speakers was not significant. Intermediate learners were not significantly more native-like than beginners (*t* (10.49) = 1.2, *p* = .256). Furthermore, the Emphasis * Proficiency interaction in the omnibus ANOVA was no longer significant when native speakers were removed (*F* (1, 12) = 1.67, *p* = .221). These results indicate that learners and beginners both differed significantly from native speakers, but not from each other.

Resolving the interaction Emphasis * Length * Vowel * Manner * Proficiency by Manner showed that, for the stops, there was a marginal interaction of Emphasis * Proficiency (*F* (1, 16) = 6.68, *p* = .008).^5^ For fricatives, there were significant to marginal interactions of Emphasis * Length * Vowel * Proficiency (*F* (4, 32) = 4.23, *p* = .023), Emphasis * Vowel * Proficiency (*F* (4, 32) = 2.77, *p* = .081), and Emphasis * Proficiency (*F* (2, 16) = 3.16, *p* = .07).^6^ These higher-order interactions are beyond the scope of this thesis.

As for the relationship between emphasis and vowel, stops and fricatives each showed interactions of Emphasis * Length * Vowel (stops: *F* (2, 32) = 7.89, *p* = .004; fricatives: *F* (2, 32) = 4.86, *p* = .031). They also each showed interactions of Emphasis * Vowel (stops: *F* (2, 32) = 8.33, *p* = .003; fricatives: *F* (2, 32) = 4.86, *p* = .031). To resolve these interactions, the absolute value of the emphasis effect (plain – emphatic) was calculated for each cell and pairwise comparisons across vowels were made using t-tests. For stops, /a/ had a larger effect of emphasis (F1 plain = 700 Hz, F1 emphatic = 662 Hz) than /u/ (F1 plain = 366 Hz, F1 emphatic = 385 Hz). This effect was significant, *t* (18) = 2.67, *p* = .016. There was also a larger effect of emphasis for /a/ than /i/ (F1 plain = 371 Hz, F1 emphatic = 372 Hz). Again, the effect reached significance, *t* (18) = 3.2, *p* = .005. However, /u/ and /i/ did not significantly differ, *t* (18) = 0.79, *p* = .441. The same was true for fricatives, /a/ had a larger effect of emphasis (F1 plain = 675 Hz, F1 emphatic = 659 Hz) than /u/ (F1 plain = 369 Hz, F1 emphatic 378 Hz). This effect was significant, *t* (18) = 2.72, *p* = .014. There was also a larger effect of emphasis for /a/ than /i/ (F1 plain = 363 Hz, F1 emphatic = 373 Hz), *t* (18) = 2.84, *p* = .011. However, /u/ and /i/ did not significantly differ, *t* (18) = 0.39, *p* = .699). The Emphasis * Length * Vowel interaction was solved in the same way. For long vowels, the emphasis effect was significantly larger for /a/ (F1 plain = 735 Hz, F1 emphatic = 679 Hz) than for /u/ (F1 plain = 341 Hz, F1 emphatic = 354 Hz), *t* (18) = 21.87, *p* < .001. There was also a larger effect of emphasis for /a/ than /i/ (F1 plain = 309 Hz, F1 emphatic = 429 Hz), *t* (18) = 18.07, *p* < .001. Moreover, the emphasis effect for /u/ was larger than that for /i/ (*t* (18) = 4.14, *p* = .001). For the short vowels, the emphasis effect for /a/ (F1 plain = 640 Hz, F1 emphatic = 642 Hz) was numerically smaller than that for /u/ (F1 plain = 394 Hz, F1 emphatic = 410 Hz), but the difference was not significant, *t* (18) = −1.51, *p* = .150. The emphasis effect for /i/ (F1 plain = 425 Hz, F1 emphatic = 429 Hz) was smaller than that for both /a/, *t* (18) = 3.05, *p* = .007, and /u/, *t* (18) = 5.29, *p* < .001.

### 3.4 Second formant frequency (F2)

F2 was analyzed using a mixed analysis of variance (ANOVA) with the within-subjects factors Emphasis (2 levels: plain, emphatic), Vowel Length (2 levels: long, short), Vowel Context (3 levels: /a/, /i/, /u/), Manner (2 levels: stop, fricative), and Voicing (2 levels: voiced, voiceless), and the between-subjects factor Proficiency (3 levels: native, intermediate, beginner). The results of the omnibus ANOVA are shown in Table 4 in Appendix B.

**Fig. 3:**
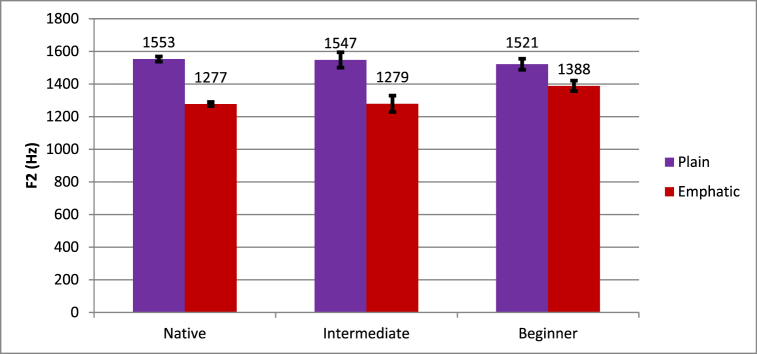
Differences in plain and emphatic F2 across levels of proficiency.

The Emphasis * Proficiency interaction reached significance. The nature of this interaction is shown in Fig. 3, indicating that native speakers showed lower F2 for emphatic (1277 Hz) than plain (1553 Hz) tokens, and intermediate learners showed lower F2 for emphatic (1279 Hz) than plain (1547 Hz) tokens. However, beginners showed a similar but smaller affect with lower F2 for emphatic (1388 Hz) than plain (1521 Hz) tokens.

To test how different each group of speakers was from native speakers, difference scores were calculated according to the procedure described above. An independent samples *t*-test showed that the difference between beginners and intermediate learners from native speakers was marginally significant; beginners tended to be less native-like than intermediate learners (*t* (10.041) = 2.003, *p* = .073).

**Fig. 4:**
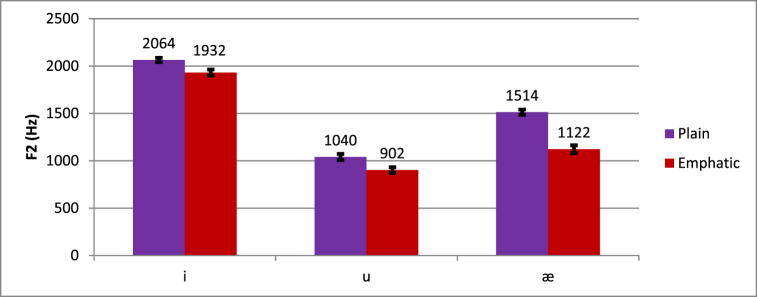
Differences in plain and emphatic F2 across vowel types.

As shown in Fig. 4, the effect of emphasis seems larger for the vowel /a/ (F2 plain = 1514 Hz, F2 emphatic = 1122 Hz) than /i/ (F2 plain = 2064 Hz, F2 emphatic = 1932 Hz) or /u/ (F2 plain = 1040 Hz, F2 emphatic 902 Hz). The effect of emphasis was significantly larger for /a/ than for /u/ (*t* (18) = 6.279, *p* < .001) and for /i/ (*t* (18) = 5.293, *p* < .001). However, /i/ and /u/ did not significantly differ from each other (*t* (18) = 0.146, *p* = .886). As for the higher-order interactions, they are beyond the scope of this study.

### 3.5 Third formant frequency (F3)

F3 was analyzed using a mixed analysis of variance (ANOVA) with the within-subjects factors Emphasis (2 levels: plain, emphatic), Vowel Length (2 levels: long, short), Vowel Context (3 levels: /a/, /i/, /u/), Manner (2: stop, fricative), and Voicing (2 levels: voiced, voiceless), and the between-subjects factor Proficiency (3 levels: native, intermediate, beginner). The results of the omnibus ANOVA are shown in Table 5 in Appendix B.

**Table 5:** Results of the omnibus ANOVA for F3.

Effect	F3
Proficiency	*F* (2, 16) = 1.24. *P* = .315
Emphasis	*F* (1,16) = 6.24, *P* = .024**
Emphasis * Proficiency	*F* (2,16) = 2.46, *P* = .117
Length	*F* (1,16) = 7.64, *P* = .014**
Length * Proficiency	*F* (2,16) = 2.84, *P* = .88*
Vowel	*F* (2,32) = 34.14, *P* < .001***
Vowel * Proficiency	*F* (4,32) = 6.03, *P* < .001***
Manner	*F* (1,16) = 3.58, *P* = .077*
Manner * Proficiency	*F* (2,16) = 2.30, *P* = .133
Voice	*F* (1,16) = 13.85, *P* = .002**
Voice * Proficiency	*F* (2,16) = .53, *P* = .596
Emphasis * Length	*F* (1,16) = .49, *P* = .493
Emphasis * Length * Proficiency	*F* (2,16) = .56, *P* = .584
Emphasis * Vowel	*F* (2,32) = 11.83, *P* < .001***
Emphasis * Vowel * Proficiency	*F* (4,32) = 1.76, *P* = .162
Length * Vowel	*F* (2,32) = 23.81, *P* < .001***
Length * Vowel * Proficiency	*F* (4,32) = 5.04, *P* = .004**
Emphasis * Length * Vowel	*F* (2,32) = 2.24, *P* = .123
Emphasis * Length * Vowel * Proficiency	*F* (4,32) = 1.45, *P* = .243
Emphasis * Manner	*F* (1,16) = 4.72, *P* = .045**
Emphasis * Manner * Proficiency	*F* (2,16) = 2.92, *P* = .083*
Length * Manner	*F* (1,16) = 1.81, *P* = .198
Length * Manner * Proficiency	*F* (2,16) = .67, *P* = .527
Emphasis* Length * Manner	*F* (1,16) = 2.15, *P* = .162
Emphasis * Length * Manner * Proficiency	*F* (2,16) = .30, *P* = .745
Vowel * Manner	*F* (2,32) = 2.19, *P* = .134
Vowel * Manner * Proficiency	*F* (4,32) = 1.45, *P* = .246
Emphasis * Vowel * Manner	*F* (2,32) = .51, *P* = .600
Emphasis * Vowel * Manner * Proficiency	*F* (4,32) = 1.38, *P* = .265
Length * Vowel * Manner	*F* (2,32) = .06, *P* = .943
Length * Vowel * Manner * Proficiency	*F* (4,32) = .655, *P* = .619
Emphasis * Length * Vowel * Manner	*F* (2,32) = 4.00, *P* = .028**
Emphasis * Length * Vowel * Manner * Proficiency	*F* (4,32) = .57, *P* = .685
Emphasis * Voice	*F* (1,16) = .54, *P* = .475
Emphasis * Voice * Proficiency	*F* (2,16) = .86, *P* = .443
Length * Voice	*F* (1,16) = 5.71, *P* = .030**
Length * Voice * Proficiency	*F* (2,16) = 3.82, *P* = .044**
Emphasis * Length * Voice	*F* (1,16) = .46, *P* = .510
Emphasis * Length * Voice * Proficiency	*F* (2,16) = .689, *P* = .517
Vowel * Voice	*F* (2,32) = 3.24, *P* = .052*
Vowel * Voice * Proficiency	*F* (4,32) = .87, *P* = .494
Emphasis * Vowel * Voice	*F* (2,32) = 1.61, *P* = .215
Emphasis * Vowel * Voice * Proficiency	*F* (4,32) = .33, *P* = .855
Length * Vowel * Voice	*F* (2,32) = .95, *P* = .381
Length * Vowel * Voice * Proficiency	*F* (4,32) = 1.39, *P* = .267
Emphasis * Length * Vowel * Voice	*F* (2,32) = 2.69, *P* = .083*
Emphasis * Length * Vowel * Voice * Proficiency	*F* (4,32) = .947, *P* = .448
Manner * Voice	*F* (1,16) = .19, *P* = .670
Manner * Voice * Proficiency	*F* (2,16) = .61, *P* = .554
Emphasis * Manner * Voice	*F* (1,16) = .23, *P* = 642
Emphasis * Manner * Voice * Proficiency	*F* (2,16) = .53, *P* = .598
Length * Manner * Voice	*F* (1,16) = .02, *P* = .886
Length * Manner * Voice * Proficiency	*F* (2,16) = 1.19, *P* = .331
Emphasis * Length * Manner * Voice	*F* (1,16) = .09, *P* = .772
Emphasis * Length * Manner * Voice * Proficiency	*F* (2,16) = 3.27, *P* = .065*
Vowel * Manner * Voice	*F* (2,32) = 1.43, *P* = .255
Vowel * Manner * Voice * Proficiency	*F* (4,32) = .59, *P* = .669
Emphasis * Vowel * Manner * Voice	*F* (2,32) = .88, *P* = .419
Emphasis * Vowel * Manner * Voice * Proficiency	*F* (4,32) = .46, *P* = .757
Length * Vowel * Manner * Voice	*F* (2,32) = 1.17, *P* = .324
Length * Vowel * Manner * Voice * Proficiency	*F* (4,32) = 1.11, *P* = .365
Emphasis * Length * Vowel * Manner * Voice	*F* (2,32) = 4.11, *P* = .040**
Emphasis * Length * Vowel * Manner * Voice * Proficiency	*F* (4,32) = 1.29, *P* = .300

* 0.1 > *p* > .05; **p < .05; ***p < .001.

**Fig. 5:**
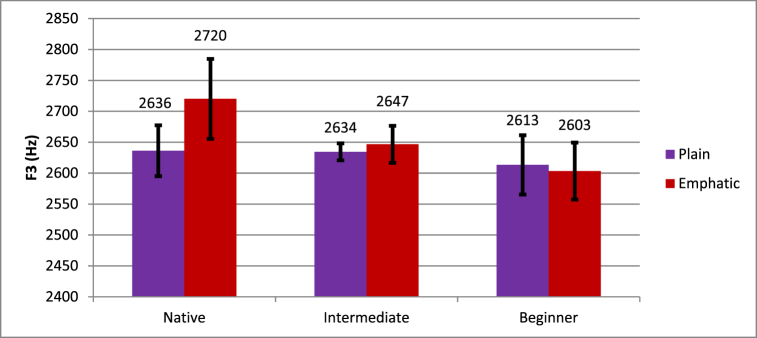
Differences in plain and emphatic F3 in the context of stops.

**Fig. 6:**
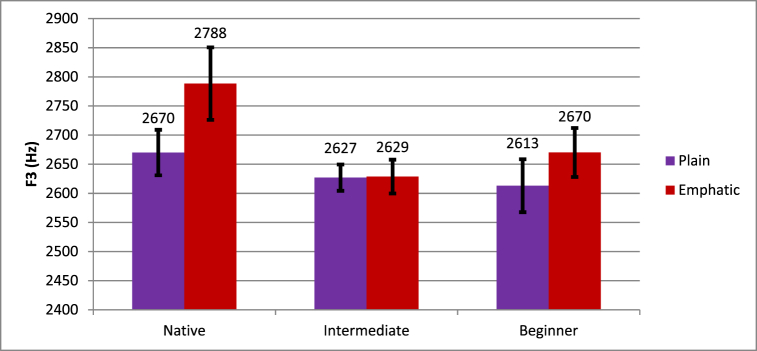
Differences in plain and emphatic F3 in the context of fricatives.

The Emphasis * Proficiency interaction did not reach significance, but the Emphasis * Manner * Proficiency did, suggesting that there may have been a relationship between emphasis and proficiency in just the stops or just the fricatives. The nature of this three-way interaction is shown in Fig. 5, Fig. 6, suggesting that for stops, native speakers showed lower F3 for plain (2636 Hz) than for emphatic (2720 Hz) stops whereas learners showed little effect of emphasis; beginning learners showed higher F3 for plain (2613 Hz) than for emphatic (2603) stops, and intermediate learners showed lower F3 for plain (2634 Hz) than for emphatic (2647 Hz) stops. For fricatives, native speakers showed much lower F3 for plain (2670 Hz) than emphatic (2788 Hz) fricatives, beginners showed somewhat lower F3 for plain (2613 Hz) than emphatic (2670 Hz) fricatives, and intermediate learners showed little difference between plain (2627 Hz) and emphatic (2629 Hz) fricatives. The interaction was resolved by Manner, revealing that there was not a significant Emphasis * Proficiency interaction for stops (*F* (2, 16) = 2.08, *p* = .158) but there was a marginal Emphasis * Proficiency interaction for fricatives (*F* (2, 16) = 2.95, *p* = .081). Plain fricatives had significantly higher F3 for native speakers (*t* (4) = −3.21, *p* = .033) and beginners (*t* (6) = −2.76, *p* = .033), but not for intermediate learners (*t* (6) = −0.05, *p* = .964).

**Fig. 7:**
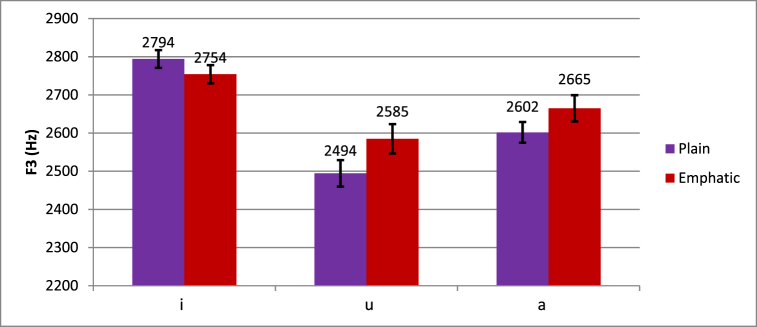
Differences in plain and emphatic F3 across vowel types.

As shown in Fig. 7 below, the effect of emphasis seems larger for the vowel /a/ (plain F3 = 2602 Hz, emphatic F3 = 2665 Hz) and /u/ (plain F3 = 2494 Hz, emphatic F3 = 2585 Hz) than for /i/ (plain F3 = 2794 Hz, emphatic F3 = 2754 Hz). For /a/, plain F3 was marginally lower than emphatic F3 (*t* (18) = -1.971, *p* = .064). For /u/, plain F3 was significantly lower than emphatic F3 (*t* (18) = -4.164, *p* = .001). As for /i/, plain F3 was marginally higher than emphatic F3 (*t* (18) = 2.042, *p* = .056). The higher-order interactions are beyond the scope of this study.

## 4 Discussion and conclusions

The results of the L2 learners are compared to those of [19], and they are also discussed within the framework of SLM developed by Ref. [20]. It is worth mentioning here that SLM makes clear predictions about the perception and production of L2 speech by highly-experienced L2 learners who have spoken their L2 for many years. However, in this study, SLM is used to account for the production of L2 speech by two groups differing in their experience with L2; these are a group of intermediate L2 learners and a group of beginning L2 learners. Although the L2 learners of varying experience had been studying Arabic for no longer than 4 years, SLM can still be used to generate predictions about learning in progress; the more proficient group is expected to produce values that are closer to those of the native speakers than the less experienced/proficient group.

According to the second hypothesis of the SLM (H2), the likelihood of forming a new phonetic category is greater when an L2 sound is judged to be perceptually distant from the closest L1 sound than when an L2 sound is judged to be perceptually close to an L1 sound. L2 research provided empirical evidence that (adult) L2 learners need many years to establish new phonetic categories for L2 sounds that are perceptually distinct from the closest L1 sounds. For example [22], reported that Japanese speakers established new phonetic categories for the English liquids /ɹ/ and /l/ after living 21 years in the US; however, the Japanese speakers who had lived for 2 years in the US at the time of the study did not show evidence of forming phonetic categories for the English liquids.

The present results indicated that L2 learners produced comparable VOT values to those of native speakers; neither the intermediate nor the beginners were different from native speakers in terms of the production of the VOT correlates of emphasis. In terms of SLM, this suggests that the L2 learners were able to detect at least some of the perceptual differences between the Arabic /tˤ/ and the perceptually closest L1 and L2 sounds, i.e., English and Arabic /t/. Therefore, it is likely that the L2 learners had established a new phonetic category for the Arabic /tˤ/. Because the results showed that COG was not a reliable acoustic correlate of emphasis (natives did not differentiate between fricatives in terms of COG), it appears that the distinction between plain and emphatic fricatives is not based on COG. This suggests that the distinction might be in the acoustic correlates carried by the following vowel. While [19] did not look at the production of VOT and COG by L2 learners of Arabic, this study provided more detailed information about the production of VOT and COG by L2 learners of Arabic who are native speakers of American English.

As for F1, the results indicated that while native speakers produced a significantly higher F1 in the emphatic context, the L2 learners showed the opposite effect; they produced a lower F1 in the emphatic environment. However, the intermediate and beginning learners were not different from each other on the F1 measure. This supports the findings of [19] in that L2 learners did not produce a significantly different emphatic F1. Concerning F2, the results showed that all the L2 learners produced a lower F2 in the emphatic context. Again, this supports the findings of [19] in that all language groups produced a lower F2 in the emphatic environment. However, the present study provided more information about the effects of proficiency, i.e., the intermediate L2 learners tended to be more native-like than the beginners on the F2 measure.

[19] found that the distinction between Arabic and English vowels is largely based on F2. This might explain why the L2 learners in the present study differed significantly from native speakers in terms of the F1 values. Previous studies also showed that L2 learners adopt different strategies and attend to certain acoustic information to categorize L2 speech sounds [23]. reported that the North German and American English vowel space have various patterns of spectral resemblance [23]. reported that where midlong and high-mid short vowels overlapped in F1, these vowels were discriminated using F2. In another study [24], found that Japanese learners relied on duration distinctions to distinguish English /ɹ/ from /l/; English speakers, on the other hand, relied on F2 and F3. Therefore, the L2 learners of Arabic might have relied on F2 as a more robust acoustic cue for the Arabic-English vowel distinction. In terms of F3, the beginning and intermediate learners patterned differently; only beginners and native speakers produced a significantly higher F3 in the emphatic context of fricatives. All groups produced numerically higher F3 in the emphatic context of stops. Again [19], did not look at the production of F3 by L2 learners of Arabic. Therefore, this study provided more information about the production of F3 by L2 learners of Arabic who are native speakers of American English. Overall, the results are in line with those of [26,27] in that adult L2 learners are capable of establishing phonetic categories for new L2 sounds. Since the intermediate and beginning learners were marginally different on the F2 measure, this suggests that the intermediate learners were a somewhat more successful in achieving target-like categorical representation of the Arabic vowels in the emphatic context. This corroborates the claim of the SLM that more experience in L2 results in more accurate production of L2.

The results of F2 showed that the effect of emphasis was larger for /a/ than /i/ and /u/. However, /i/ and /u/ did not differ from each other with regard to the effect of emphasis. The results of F3 showed that the relationship between emphasis and proficiency depended on whether the preceding consonant was a stop or fricative. In the context of stops, beginning learners produced a numerically higher F3 in the plain context, and intermediate learners produced a numerically lower F3 in the plain context. In the context of fricatives, only beginning learners produced a higher F3 in the emphatic context, whereas the intermediate learners produced a numerically higher F3 in the emphatic context. This is in line with the findings of a number of previous studies (e.g., Refs. [[6], [7], [8],^10^,[16], [17], [18]]).

This study provided a more complete understanding of the linguistic behavior of L2 learners of Arabic than that provided by Ref. [19]. The acoustic cues that were examined in Ref. [19] were only F1 and F2 of the vowels in the context of emphatic and plain consonants. In addition, only language experience and vowel pharyngealization were the independent variables in the statistical analyses of the data. The present study was more comprehensive in terms of the acoustic cues that were examined; in addition to measuring F1–F3 values in emphatic and plain environments, the characteristics of the consonants were also examined by measuring the VOT of emphatic and plain stops and COG of emphatic and plain fricatives. More independent variables were also used; these are vowel quality, vowel quantity, manner of articulation, and voicing.

The present acoustic findings raise the question about how the learners’ productions are perceived by native Arab listeners. Future perception experiments are planned to determine if learners’ emphatic productions are indeed perceived as emphatic. Our acoustic results suggest this may be the case for the intermediate learners but not for the beginning learners. By relating the perceptual results to the acoustic measurements, we will also be able to determine the extent to which different acoustic properties contribute to the perception of emphasis.

The pedagogical implications that can be drawn from the above arguments is that explicit phonetic instruction, including appropriate types of exposure, practice, and feedback should improve the L2 learners’ perception and production of the target sounds. In other words, language teachers should help L2 learners precisely attune their perceptive skills, and this attunement would facilitate later development in production, i.e., adding or modifying sensorimotor programs for producing sounds in L2. Therefore, it is suggested that pronunciation instruction materials include more exercises at the production level when the target sound presents more difficulties in production than in perception (as is the case with Arabic emphatic consonants). Including more production exercises throughout the teaching materials will offer further practice with production skills [10].

## Author contribution statement

Hesham Aldamen: Conceived and designed the experiments; Performed the experiments; Analyzed and interpreted the data; Contributed reagents, materials, analysis tools or data; Wrote the paper.

Mutasim Al-Deaibes: Performed the experiments; Analyzed and interpreted the data; Wrote the paper.

## Funding statement

This research did not receive any specific grant from funding agencies in the public, commercial, or not-for-profit sectors.

## Data availability statement

The authors do not have permission to share data.

## Declaration of interest’s statement

The authors declare no competing interests.

## Figures and Tables

**Table 3:** Results of the omnibus ANOVA for F1.

Effect	F1
Proficiency	*F* (2,16) = 2.24*, p* = .139
Emphasis	*F* (1,16) = 0.01, *p* = .913
Emphasis * Proficiency	*F* (2,16) = 6.38, *p* = .009**
Length	*F* (1,16) = 20.45, *p* < .001***
Length * Proficiency	*F* (2,16) = 0.4, *p* = .677
Vowel	*F* (2,32) = 507.14, *p* < .001***
Vowel * Proficiency	*F* (4,32) = 2.06, *P* = .141
Manner	*F* (1,16) = 1.93, *P* = .184
Manner * Proficiency	*F* (2,16) = .37, *P* = .697
Voice	*F* (1,16) = 9.93, *P* = .006**
Voice * Proficiency	*F* (2,16) = 2.08, *P* = .157
Emphasis * Length	*F* (1,16) = 7.77, *P* = .013**
Emphasis * Length * Proficiency	*F* (2,16) = .58, *P* = .571
Emphasis * Vowel	*F* (2,32) = 5.91, *P* = .017**
Emphasis * Vowel * Proficiency	*F* (4,32) = 1.90, *P* = .165
Length * Vowel	*F* (2,32) = 84.76, *P* < .001***
Length * Vowel * Proficiency	*F* (4,32) = 1.84, *P* = .167
Emphasis * Length * Vowel	*F* (2,32) = 8.60, *P* = .004**
Emphasis * Length * Vowel * Proficiency	*F* (4,32) = 1.34, *P* = .286
Emphasis * Manner	*F* (1,16) = .70, *P* = .415
Emphasis * Manner * Proficiency	*F* (2,16) = .95, *P* = .408
Length * Manner	*F* (1,16) = .03, *P* = .874
Length * Manner * Proficiency	*F* (2,16) = 1.41, *P* = .274
Emphasis* Length * Manner	*F* (1,16) = 1.02, *P* = .328
Emphasis * Length * Manner * Proficiency	*F* (2,16) = .26, *P* = .772
Vowel * Manner	*F* (2,32) = .70, *P* = .464
Vowel * Manner * Proficiency	*F* (4,32) = 1.089, *P* = .372
Emphasis * Vowel * Manner	*F* (2,32) = 2.70, *P* = .095*
Emphasis * Vowel * Manner * Proficiency	*F* (4,32) = 1.01, *P* = .409
Length * Vowel * Manner	*F* (2,32) = .67, *P* = .488
Length * Vowel * Manner * Proficiency	*F* (4,32) = 2.03, *P* = .132
Emphasis * Length * Vowel * Manner	*F* (2,32) = 2.12, *P* = .138
Emphasis * Length * Vowel * Manner * Proficiency	*F* (4,32) = 2.86, *P* = .04**
Emphasis * Voice	*F* (1,16) = .44, *P* = .515
Emphasis * Voice * Proficiency	*F* (2,16) = .74, *P* = .492
Length * Voice	*F* (1,16) = 1.08, *P* = .314
Length * Voice * Proficiency	*F* (2,16) = .31, *P* = .739
Emphasis * Length * Voice	*F* (1,16) = 3.45, *P* = .082*
Emphasis * Length * Voice * Proficiency	*F* (2,16) = .74, *P* = .491
Vowel * Voice	*F* (2,32) = 1.60, *P* = .217
Vowel * Voice * Proficiency	*F* (4,32) = 1.30, *P* = .291
Emphasis * Vowel * Voice	*F* (2,32) = .383, *P* = .642
Emphasis * Vowel * Voice * Proficiency	*F* (4,32) = .537, *P* = .675
Length * Vowel * Voice	*F* (2,32) = .39, *P* = .654
Length * Vowel * Voice * Proficiency	*F* (4,32) = 2.40, *P* = .081*
Emphasis * Length * Vowel * Voice	*F* (2,32) = .002, *P* = .994
Emphasis * Length * Vowel * Voice * Proficiency	*F* (4,32) = .98, *P* = .424
Manner * Voice	*F* (1,16) = 3.74, *P* = .071*
Manner * Voice * Proficiency	*F* (2,16) = .97, *P* = .400
Emphasis * Manner * Voice	*F* (1,16) = .24, *P* = .631
Emphasis * Manner * Voice * Proficiency	*F* (2,16) = 1.01, *P* = .631
Length * Manner * Voice	*F* (1,16) = .86, *P* = .369
Length * Manner * Voice * Proficiency	*F* (2,16) = .14, *P* = .870
Emphasis * Length * Manner * Voice	*F* (1,16) = 4.99, *P* = .040**
Emphasis * Length * Manner * Voice * Proficiency	*F* (2,16) = .34, *P* = .718
Vowel * Manner * Voice	*F* (2,32) = .51, *P* = .596
Vowel * Manner * Voice * Proficiency	*F* (4,32) = 1.32, *P* = .284
Emphasis * Vowel * Manner * Voice	*F* (2,32) = 1.29, *P* = .287
Emphasis * Vowel * Manner * Voice * Proficiency	*F* (4,32) = .24, *P* = .889
Length * Vowel * Manner * Voice	*F* (2,32) = 1.63, *P* = .217
Length * Vowel * Manner * Voice * Proficiency	*F* (4,32) = .93, *P* = .448
Emphasis * Length * Vowel * Manner * Voice	*F* (2,32) = .12, *P* = .831
Emphasis * Length * Vowel * Manner * Voice * Proficiency	*F* (4,32) = 1.54, *P* = .229

*1 > *p* > .05; **p < .05; ***p < .001.

**Table 4:** Results of the omnibus ANOVA for F2.

Effect	F2
Proficiency	*F* (2,16) = .52, *p* = .607
Emphasis	*F* (1,16) = 108.99, *P* < .001***
Emphasis * Proficiency	*F* (2,16) = 4.91, *P* = .022**
Length	*F* (1,16) = 46.56, *p* < .001***
Length * Proficiency	*F* (2, 16) = .37, *p* = . 695
Vowel	*F* (2,32) = 534.28, *p* < .001***
Vowel * Proficiency	*F* (4,32) = 1.15, *p* = .351
Manner	*F* (1,16) = .84, *p* = .373
Manner * Proficiency	*F* (2,16) = .95, *p* = .407
Voice	*F* (1,16) = .40, *p* = .538
Voice * Proficiency	*F* (2,16) = 2.27, *p* = .136
Emphasis * Length	*F* (1,16) = 4.13, *p* = .059*
Emphasis * Length * Proficiency	*F* (2,16) = 3.38, *p* = .060*
Emphasis * Vowel	*F* (2,32) = 25.72, *p* < .001***
Emphasis * Vowel * Proficiency	*F* (4,32) = 2.10, *p* = .118
Length * Vowel	*F* (2,32) = 188.50, *p* < .001***
Length * Vowel * Proficiency	*F* (4,32) = .95, *p* = .447
Emphasis * Length * Vowel	*F* (2,32) = 1.73, *p* = .196
Emphasis * Length * Vowel * Proficiency	*F* (4,32) = 2.47, *p* = .071*
Emphasis * Manner	*F* (1,16) = .41, *p* = .533
Emphasis * Manner * Proficiency	*F* (2,16) = .29, *p* = .756
Length * Manner	*F* (1,16) = .863, *p* = .367
Length * Manner * Proficiency	*F* (2,16) = .23, *p* = .797
Emphasis* Length * Manner	*F* (1, 16) = .002, *p* = .963
Emphasis * Length * Manner * Proficiency	*F* (2,16) = .16, *p* = .855
Vowel * Manner	*F* (2,32) = .95, *p* = .382
Vowel * Manner * Proficiency	*F* (4,32) = .07, *p* = .982
Emphasis * Vowel * Manner	*F* (2,32) = .66, *p* = .488
Emphasis * Vowel * Manner * Proficiency	*F* (4,32) = .88, *p* = .469
Length * Vowel * Manner	*F* (2,32) = 2.04, *p* = .149
Length * Vowel * Manner * Proficiency	*F* (4,32) = .494, *p* = .733
Emphasis * Length * Vowel * Manner	*F* (2,32) = .58, *p* = .541
Emphasis * Length * Vowel * Manner * Proficiency	*F* (4,32) = .22, *p* = .908
Emphasis * Voice	*F* (1,16) = .17, *p* = .682
Emphasis * Voice * Proficiency	*F* (2,16) = 2.61, *p* = .104
Length * Voice	*F* (1,16) = .31, *p* = .583
Length * Voice * Proficiency	*F* (2,16) = 2.05, *p* = .161
Emphasis * Length * Voice	*F* (1,16) = 1.62, *p* = .221
Emphasis * Length * Voice * Proficiency	*F* (2,16) = .022, *p* = .978
Vowel * Voice	*F* (2,32) = .02, *p* = .978
Vowel * Voice * Proficiency	*F* (4,32) = .95, *p* = .443
Emphasis * Vowel * Voice	*F* (2,32) = .40, *p* = .668
Emphasis * Vowel * Voice * Proficiency	*F* (4,32) = .60, *p* = .659
Length * Vowel * Voice	*F* (2,32) = 1.51, *p* = .238
Length * Vowel * Voice * Proficiency	*F* (4,32) = .19, *p* = .934
Emph * Length * Vowel * Voice	*F* (2,32) = .53, *p* = .580
Emphasis * Length * Vowel * Voice * Proficiency	*F* (4,32) = .50, *p* = .721
Manner * Voice	*F* (1,16) = 1.33, *p* = .266
Manner * Voice * Proficiency	*F* (2,16) = .17, *p* = .849
Emphasis * Manner * Voice	*F* (1,16) = 1.50, *p* = .240
Emphasis * Manner * Voice * Proficiency	*F* (2,16) = .69, *p* = .515
Length * Manner * Voice	*F* (1,16) = .13, *p* = .722
Length * Manner * Voice * Proficiency	*F* (2,16) = 1.05, *p* = .372
Emphasis * Length * Manner * Voice	*F* (1,16) = .82, *p* = .379
Emphasis * Length * Manner * Voice * Proficiency	*F* (2,16) = 2.23, *p* = .140
Vowel * Manner * Voice	*F* (2,32) = 3.61, *p* = .040**
Vowel * Manner * Voice * Proficiency	*F* (4,32) = .85, *p* = .502
Emphasis * Vowel * Manner * Voice	*F* (2,32) = .23, *p* = .712
Emphasis * Vowel * Manner * Voice * Proficiency	*F* (4,32) = .61, *p* = .603
Length * Vowel * Manner * Voice	*F* (2,32) = .39, *p* = .670
Length * Vowel * Manner * Voice * Proficiency	*F* (4,32) = 1.93, *p* = .134
Emphasis * Length * Vowel * Manner * Voice	*F* (2,32) = .14, *p* = .849
Emphasis * Length * Vowel * Manner * Voice * Proficiency	*F* (4,32) = .97, *p* = .432

* 0.1 > *p* > .05; **p < .05; ***p < .001.

**Table:** 

Word (Plain)	Gloss	Word (emphatic)	Gloss
ta:b	He repented.	tˤa:b	He recovered.
da:l	Letter D	dˤa:l	Astray
sa:d	He dominated.	sˤa:d	He hunted.
ða:b	Melted.	ðˤa:b	He is keeping.
tu:b	Repent!	tˤu:b	Brick
du:ʔ	Taste!	dˤu:ʔ	Light
su:r	Fence	sˤu:r	Name of a city
ðu:b	Melt!	ðˤu:b	Nonword
ti:n	Figs	tˤi:n	Mud
di:f	Nonword	dˤi:f	Add!
si:b	Leave!	sˤi:b	Touch!
ði:b	Wolf	ðˤi:b	Nonword
tab	He definitely lost!	tˤab	He arrived.
dab	He dropped.	dˤab	A desert animal
sab	He insulted.	sˤab	He poured.
ðab	Nonword	ðˤab	He kept.
tub	Repent!	tˤub	Medicine
dub	Bear	dˤub	Keep!
sub	Insult!	sˤub	Pour!
ðub	Melt!	ðˤub	Keep!
tib	Tape	tˤib	Medicine
dib	Drop	dˤib	Nonword
sib	Insult!	sˤib	Nonword
ðil	Humiliate!	ðˤil	Shadow

## Appendix B

Stimuli

## Appendix C

Language Experience and Proficiency Questionnaire (LEAP-Q)

Last name: ______________. First name: _______________. Age: ______. Today’s Date:___________

(1) Please list all languages (including English and Arabic) you know in order of dominance.

(1)_____________ (2) _____________ (3) ________________ (4) ______________ (5) _______________

(2) Please list all languages (including English and Arabic) you know in order of acquisition. (Your native language first)

(1)_____________ (2) _____________ (3) ______________ (4) ______________ (5) _______________

(3) For each language you know, please list what percentage of time you are currently and on average exposed to that language. (Your percentages should add up to 100%)

List language here (1) __________ (2) __________ (3) _________ (4) ___________ (5) _____________

List percentage here __________ __________ __________ _____________ ____________

(4) Are a graduate or undergraduate student? ______________. What is your major? ________________
(5) How long have you been studying Arabic at KU? ______________________
(6) Is your Arabic teacher a native speaker of Arabic? _____. Where does s/he come from? ___________
(7) Do you use Arabic outside classroom? _____. If yes, please specify how: _________________________________________________________________________________________
(8) Have you lived in an Arabic-speaking country? ________. What is it? __________. How long? _____________. Have you studied Arabic there? _______. How long? _______________.
(9) Have you studied Arabic elsewhere, besides what you have mentioned above? ______. If yes, please specify: __________________________________________________________________________________________
(10) Please identify your level of proficiency in Arabic. Draw a circle around one of the four options!

Speaking:very goodsomewhat goodso-sosomewhat poorvery poor

Listening:very goodsomewhat goodso-sosomewhat poorvery poor

Reading:very goodsomewhat goodso-sosomewhat poorvery poor

Writing:very goodsomewhat goodso-sosomewhat poorvery poor

## Footnotes

1 According to Ref. [5], F2 of the vowel after the emphatic sound undergoes a sudden sharp decrease then gradually starts to rise as it goes far in distance from emphatic sound (pp. 96–100).

2 That the participants of the study had learned Arabic for only a few years, this could be one of the limitations of the study. Future studies should recruit participants who had been studying Arabic for a longer period of time.

3 Degrees of freedom for this test are adjusted because equal variances were not assumed.

4 For this graph and all subsequent graphs, error bars represent standard errors.

5 There were also several significant or marginal effects that did not include Emphasis * Proficiency or Emphasis * Vowel interactions. These were as follows: Length * Vowel (*F* (2, 32) = 61.2, *p* < .001), Vowel * Proficiency, (*F* (4, 32) = 2.49, *p* = .092) Vowel (*F* (2, 32) = 461.68, *p* < .001), Length (*F* (1, 16) = 21.64, *p* < .001).

6 There were also several significant or marginal effects that did not include Emphasis * Proficiency interactions. These were as follows: Emphasis * Length (*F* (1, 16) = 9.92, *p* = .006), Vowel (*F* (2, 32) = 403.71, *p* < .001), Length (*F* (1, 16) = 14.04, *p* = .002).
